# Usability and Acceptability of a Home Blood Pressure Telemonitoring Device Among Community-Dwelling Senior Citizens With Hypertension: Qualitative Study

**DOI:** 10.2196/10975

**Published:** 2018-07-24

**Authors:** Lauren Albrecht, Peter W Wood, Miriam Fradette, Finlay A McAlister, Doreen Rabi, Pierre Boulanger, Raj Padwal

**Affiliations:** ^1^ Department of Pediatrics University of Alberta Edmonton, AB Canada; ^2^ Department of Medicine University of Alberta Edmonton, AB Canada; ^3^ Departments of Medicine, Community Health and Cardiac Sciences University of Calgary Calgary, AB Canada; ^4^ Department of Computing Science University of Alberta Edmonton, AB Canada

**Keywords:** blood pressure, telemonitoring, community-dwelling, qualitative

## Abstract

**Background:**

Hypertension is a major cause of cardiovascular disease in older individuals. To ensure that blood pressure (BP) levels are within the optimal range, accurate BP monitoring is required. Contemporary hypertension clinical practice guidelines strongly endorse the use of home BP measurement as a preferred method of BP monitoring for individuals with hypertension. The benefits of home BP monitoring may be optimized when measurements are telemonitored to care providers; however, this may be challenging for older individuals with less technological capabilities.

**Objective:**

The objective of this qualitative study was to examine the usability and acceptability of a home BP telemonitoring device among senior citizens.

**Methods:**

We conducted a qualitative descriptive study. Following a 1-week period of device use, individual, semistructured interviews were conducted. Interview audio recordings were anonymized, de-identified, and transcribed verbatim. We performed thematic analysis on interview transcripts.

**Results:**

Seven senior citizens participated in the usability testing of the home BP telemonitoring device. Participants comprised females (n=4) and males (n=3) with a mean age of 86 years (range, 70-95 years). Overall, eight main themes were identified from the interviews: (1) positive features of the device; (2) difficulties or problems with the device; (3) device was simple to use; (4) comments about wireless capability and components; (5) would recommend device to someone else; (6) would use device in future; (7) suggestions for improving the device; and (8) assistance to use device. Additional subthemes were also identified.

**Conclusions:**

Overall, the home BP telemonitoring device had very good usability and acceptability among community-dwelling senior citizens with hypertension. To enhance its long-term use, few improvements were noted that may mitigate some of the relatively minor challenges encountered by the target population.

## Introduction

Population aging is occurring in countries across the world at a rate that by 2050, nearly 25% of the global population is expected to be over the age of 60 years [1]. Advanced age is often complicated by cardiovascular morbidity, which frequently leads to disability and death [2]. Hypertension is a major cause of cardiovascular disease in older individuals [3,4], but senior citizens also derive greater benefits from treatment of elevated blood pressure (BP) than younger patients [5,6]. The management of hypertension in senior citizens can be challenging and must balance the risk of treatment-related serious complications (including hypotension, postural dizziness, syncope, falls, and metabolic side effects) [3,7-9] against the well-established cardiovascular benefits associated with BP lowering.

To ensure that BP levels are within the optimal range, accurate BP monitoring is required. Contemporary hypertension clinical practice guidelines strongly endorse the use of home BP measurement as a preferred method of BP monitoring for individuals with hypertension [10]. The benefits of home BP monitoring may be optimized when measurements are telemonitored to care providers [11,12]. BP telemonitoring involves the electronic and secure transmission of BP readings in real time to a central electronic health care portal, with summarized BP data presented to patients and providers. Through process automation and protocolization, BP telemonitoring can ensure that home BP monitoring is performed in a guideline-concordant manner, ensuring the proper frequency and timing of BP measurements [12]. Telemonitoring theoretically could eliminate the need for an in-person clinic visit, thereby increasing health care delivery efficiency, minimizing costs, and making the use of provider and patient time more efficient [12]. A meta-analysis of 23 randomized controlled trials (RCTs; 7037 patients) reported that home BP telemonitoring reduced BP by 5/3 mm Hg compared with usual care (*P*<0.0001 for both systolic and diastolic BP) [13]. This is a clinically important reduction in BP, as a 5-mm Hg reduction can decrease the risk of total cardiovascular disease by 17%, stroke by 18%, and myocardial infarction by 15% [14,15].

Adoption of telemonitoring will only occur if the process and technology are deemed usable and acceptable by patients and caregivers. Use of home BP telemonitoring may be particularly challenging in senior citizens, who, by virtue of being less technologically literate, may have greater difficulty using a telemonitoring device. Therefore, the purpose of this qualitative study, embedded within an ongoing RCT (trial registration Clinicaltrials.gov NCT02721667) examining the clinical and cost-effectiveness of telemonitoring among community-dwelling senior citizens with hypertension [16], was to examine the usability and acceptability of a home BP telemonitoring device within this particular patient population.

## Methods

### Study Design

A qualitative descriptive study was conducted [17,18] embedded within an RCT comparing BP telemonitoring plus pharmacist case management with usual care to optimize BP control among senior citizens residing in supportive living. Half of the study sample will receive home BP telemonitoring, with measurements telemonitored to a pharmacist case manager, who will provide guideline-concordant, protocolized antihypertensive medication adjustment. Patients in the usual care arm will receive a home BP monitor that they can use to record their BP, but neither they nor their providers will receive access to telemonitored measurements or pharmacist case management. They will be asked to follow-up with their regular care providers as needed. A protocol paper has been published [16]; the most updated protocol can be found on clinicaltrials.gov (NCT02721667). The University of Alberta Research Ethics Board approved the study.

### Study Population

Participants were community-dwelling senior citizens (aged ≥65 years) with hypertension residing in a supportive living residence. The target sample size for this qualitative study was 5-8 participants based on a prior research demonstrating that the first 4-5 users typically identify 80% of all usability problems, including the most severe usability issues; beyond this, a higher sample size has low incremental yield as data saturation has been reached [19].

### Data Collection

Participating senior citizens were instructed to use the home BP telemonitoring device and perform all measurements according to the recommended techniques for home BP measurement. To activate the device, the subject was required to initiate a BP measurement by pressing the appropriate device button. The remainder of the process was automated. Four measurements were taken daily (ie, 2 × am, 2 × pm) for 1 week [10]. Following this period of device use, participants completed individual, semistructured interviews (Multimedia Appendix 1) to obtain usability and acceptability information, probe participants’ responses, and give participants freedom to respond and illustrate concepts in an open-ended fashion [20]. The interviews were conducted in-person and were audio recorded. Audio recordings were anonymized, de-identified, and transcribed verbatim.

### Telemonitoring System

The custom-built Technomed telemonitoring system consists of 5 components: (1) a commercially available, Bluetooth-enabled oscillometric BP monitor (A&D Medical UA-651BLE, supplied with cuff size medium [23-37 cm, UA-290] or large [31-45 cm, UA-291]); (2) a data transmission hub (Lamprey Networks Inc.); (3) a Web portal interface (Telemed Diagnostic Management Inc); (4) an android-based interface for hub programming (Advanced Man Machine Interface Laboratory, University of Alberta); and (5) in patients with no existing internet access, a selectively deployed (based on home internet availability) Subscriber Identity Module (SIM)­–based internet hotspot. Prior to operation, the system is set up by connecting the hub to the internet (hotspot or home internet), assigning a patient identification using the android app, and creating a patient profile on the Web portal.

**Figure 1:**
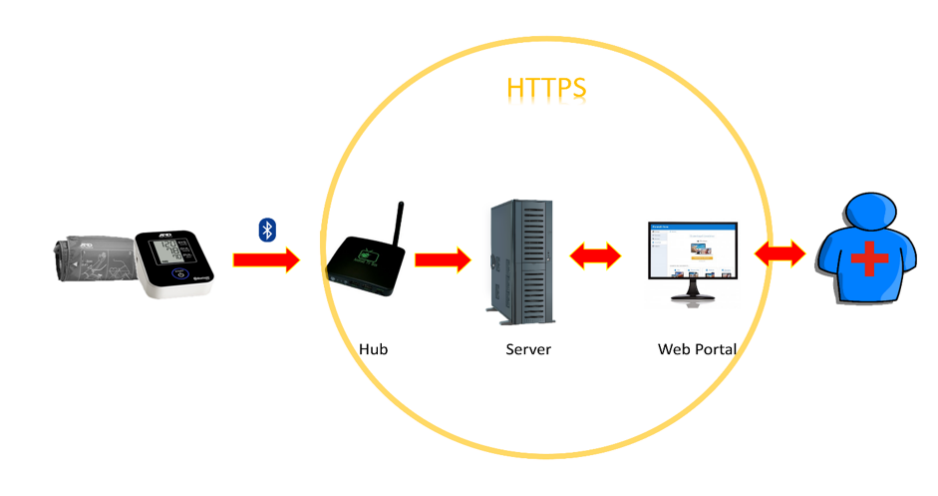
System flowchart for Technomed telemonitoring blood pressure system. HTTPS: hypertext transfer protocol secure.

### Blood Pressure Measurements

Once the system is set up, the patient is instructed to follow the 7-day series BP protocol initiated by performing a measurement using the A&D device. This measurement is sent to the Web portal via the Bluetooth-connected hub (Figure 1). The data are summarized in a variety of formats including individual readings, averages (mean of all readings minus the first day, as recommended by current guidelines) [10], and graphed values to depict temporal trends. The case manager uses these data to adjust the patient’s BP medication (uptitrate if BP is too high, downtitrate if BP too low, or leave unchanged if BP is within the optimal range).

### Data Analysis

Interview transcripts were managed and analyzed using NVivo data management software [21]. Thematic analysis was used to break interview text into small units for a detailed, nuanced account of the data [22-24]. Thematic analysis was guided by the hybrid approach of inductive and deductive coding and theme development described by Fereday & Muir-Cochrane [25]. Deductive coding of the interview transcripts was done first using the semistructured interview guide as a framework; smaller units of data that emerged inductively were coded for increased granularity and specificity.

## Results

### Sample Demographics

Seven senior citizens participated in the usability testing of the home BP telemonitoring system from November 2016 to June 2017. Participants comprised females (n=4) and males (n=3) with a mean age of 86 years (range 70-95 years). Additional demographics are summarized in Table 1. The mean baseline home systolic BP of the 7 participants was 131 mm Hg (range 99-181 mm Hg) and the mean diastolic BP was 71 mm Hg (range, 47-125 mm Hg).

### Themes

Overall, eight main themes were identified and two main themes were further classified into subthemes for increased granularity and specificity (Table 2).

**Table 1:** Participant demographics.

Participant number	Sex	Age (years)	Partnered or single	Caregiver providing assistance (yes or no)	Notes
1	Male	89	Single	No	N/A^a^
2	Female	89	Single	No	N/A
3	Female	70	Single	No	Reported visual impairment
4	Male	86	Partnered	Yes	Reported hearing impairment
5	Female	91	Single	No	Reported mild dementia
6	Female	80	Partnered, lived alone	No	N/A
7	Male	95	Single	No	N/A

^a^N/A: not applicable.

**Table 2:** Main themes and subthemes

Main themes and subthemes			Number of interviews theme is referenced in	Number of overall references for theme
**Positive features of the device**			7	35
	General positive comments about device		6	15
	**Specific positives about device**		7	20
		Cuff was appropriate	6	12
		Start button worked well	6	7
		Display screen worked well	3	3
		No bruising after device use	1	1
**Difficulties or problems with device**			7	28
	No difficulties or problems with device		2	2
	General difficulties or problems with device		1	1
	**Specific difficulties or problems with device**		7	25
		Issues positioning and securing cuff	5	8
		Cuff too large for arm	3	8
		Knowing or remembering when to take blood pressure measurements	3	7
		Error messages on display screen	2	4
		Battery issues	1	2
Device was simple to use			6	16
Comments about wireless capability and components			6	8
Would recommend device to someone else			7	8
Would use device in future			6	6
Suggestions for improving the device			5	5
Assistance to use device			1	1

**Table 3:** Specific positive features of the device.

Feature	Description	Supporting quote from interview
Cuff was of appropriate tightness	All participants described the cuff to be of appropriate tightness. It only caused minimal pain for a short duration (ie, when the cuff was fully inflated), similar to other blood pressure cuffs they had used in the past.	*Participant: No, I think it was just right.* *Interviewer: Good, okay.* *Participant: Cause it didn’t bother me that much. But it bothered me a little bit. [Laughing]* [Participant 5]
Start button worked well	Six participants noted that the start button was large enough to locate, easy to press to activate reading, and did not stick.	*But, yeah. So, other than that, it’s...it’s easy to use. The start and-and the stop. And, I mean, it’s quite simple.* [Participant 6]
Display screen worked well	Three participants described the display screen as large enough and easy to read, even for those with visual impairments.	*Participant: Um...well, I-ah, I mean you know, it’s um, it’s got a...like, I’m legally blind.* *Interviewer: Okay.* *Participant: And-and so I-I have about 12 percent vision. So I mean, for me, like it-[stammers] it’s ah-the-it’s quite clear on the-on the screen and that.* [Participant 3]
No bruising after device use	One participant noted that he or she had no bruising after using the device.	—

### Theme Descriptions

#### Positive Features of the Device

The interview guide included questions to ascertain the positive features of the device and elicit what worked for the participants. A range of answers were given. These fell into 2 subthemes: (1) general positive comments about the device and (2) specific positive features of the device.

For the first subtheme, 6 participants described the device in general positive terms, including stating it “worked well” and “was a very good set-up.” All participants provided specific feedback on positive features of the device (subtheme 2). These fell into 4 subcategories: (1) cuff was of appropriate tightness; (2) start button worked well; (3) display screen worked well; and (4) no bruising after device use. These subcategories are described in detail in Table 3.

#### Difficulties or Problems With the Device

The interview guide included questions to ascertain whether there were any difficulties or problems using the device. A range of answers were given. These fell into 3 subthemes: (1) no difficulties or problems with the device; (2) general difficulties or problems with the device; and (3) specific difficulties or problems with the device.

For the first subtheme, in response to a general question, 2 participants indicated that there were no difficulties or problems using the device. For the second subtheme, 1 participant indicated that using the device was “a little bit awkward” but that once you practice a few times, it worked well. However, all 7 participants identified specific difficulties or issues with the device (subtheme 3). These concerns fell into 5 subcategories: (1) issues positioning and securing cuff; (2) cuff too large for the arm; (3) knowing or remembering when to take BP measurements; (4) error messages on display screen; and (5) battery issues. These subcategories are described in detail in Table 4.

#### Device Was Simple to Use

All 7 participants felt that the device was simple and straightforward to use, as demonstrated by the following conversation:

Interviewer: So, did you find the device simple, to use?

Participant: Oh, yeah.

Interviewer: Yeah?

Participant: Yeah, I did. Yes.

Interviewer: And there was noth-was there anything about it that maybe was a bit tricky at first?

Participant: No, not-ah, not really. If you just followed the instructions, ah...the-all you gotta do is push the button and it-it goes, you know.

Interviewer: Yeah [laughing].

Participant: You don’t have to figure it out. It’s not like you’re doing ah...puzzle or something like that. Ah...

Interviewer: Very true.

Participant: No, not at all.

Participant 1

#### Comments About Wireless Capability and Components

Six participants commented on the wireless technology aspect of the system. Overall, the participants knew that study staff had plugged in 1 or 2 peripheral devices to assist with the operation of the BP device, but they did not know what the peripheral devices did; they did not touch these pieces during their week of using the device.

#### Would Recommend Device to Someone Else

All participants indicated that they would recommend this device to someone else if they needed regular BP monitoring.

#### Would Use Device in Future

Six participants indicated a willingness to use this device in the future if their condition required regular BP monitoring.

#### Suggestions for Improving the Device

Two participants indicated that no improvements were needed in the BP device. One participant suggested that a helpful improvement would be a signal from the device to indicate when to take a BP measurement and another indicated that some form of feedback from the device to know whether the device was positioned and secured properly would be helpful. A third improvement would be decreased tightness of the cuff, if possible.

#### Assistance to Use Device

One participant required some assistance from his or her spouse to use the device. The spouse did not assist during the morning BP readings and assisted only during the afternoon or evening readings. The spouse helped to secure and position the device for the participant because it was difficult to do so with one hand.

**Table 4:** Specific difficulties or problems using the device.

Feature	Description	Supporting quote from interview
Issues positioning and securing cuff	Five participants found the cuff difficult to position and secure on the arm. This was largely an issue of having enough dexterity to pull the fabric tight and wrap it around the arm with only one hand. Participants indicated that while pulling and fastening, the cuff would slide back and forth, and the participants were unsure if it was properly placed on the arm. Participants used the crook of the elbow as well as the dot on the cuff as helpful guides but did not feel it was optimal.	*Participant: And so, then you-[stammers] you’re trying to tighten it. And then you put your-in the meantime, you’re struggling to try and get it...* *Interviewer: I see, yeah.* *Participant: ...and so you think, “Okay, can I pull it with this? No, that’s not gonna work.” So...I don’t know how-and then, you raise your blood pressure trying to...* *Interviewer: [Laughing] I see, yes.* *Participant: To-to do that.* *Interviewer: Kind of a catch-22.* [Participant 6]
Cuff too large for arm	Three participants found that the cuff was too large and the extra material was cumbersome to manage with a single hand while securing the cuff.	*Participant: Yes. And the thing just slithered around. So it was not a good fit.* *Interviewer: Okay.* *Participant: It was a terrible fit.* *Interviewer: It was-* *Participant: A terrible choice.* *Interviewer: Too baggy?* *Participant: Oh, yeah.* *Interviewer: Yeah.* *Participant: I should have had a small cuff.* *Interviewer: Okay.* *Participant: They always use a small cuff on me at the...[chuckling] when I go to the doctor.* [Participant 2]
Knowing or remembering when to take blood pressure (BP) measurements	Five participants expressed uncertainty about knowing or remembering when to take the BP measurements. For one participant this meant forgetting to take two measurements at one time, and for the other 2 participants, this meant taking readings at different times than directed.	*Participant: Yes, but that was-trying to figure out that it was the right time.* *Interviewer: Uhum.* *Participant: You know, 10 to 12 hours apart. And I wasn’t right in getting that every time.* *Interviewer: Uhum.* *Participant: I was a little bit...too short or too long, I’m not quite sure which.* [Participant 5]
Error messages on display screen	Two participants experienced error messages on the display screen. When an error message appeared on the device, both participants attempted to take another BP reading and repeated this process until it worked and a reading was displayed, which resulted in slight confusion.	*Participant: Ah, yeah. If I had an error message, I’d just...put-push the stop but-button, and push the Start again.* *Interviewer: Okay. And that worked?* *Participant: Uhum.* *Interviewer: Okay. Well, that’s good.* *Participant: Yeah.* *Interviewer: Was it a little bit frustrating?* *Participant: Ah...oh, I was beginning to wonder if I’m doing it right.* *Interviewer: Yeah.* *Participant: Is there-is it my fault that it’s getting the error message? I don’t think so. But it might have been.* [Participant 7]
Battery issues	One participant experienced a battery issue with the device and did not know how to resolve it. The participant called study staff to resolve the issue; study staff replaced the battery, and the participant continued to use the device.	—

## Discussion

### Principal Findings

The purpose of this qualitative study, conducted concurrently with an RCT, was to assess the usability and acceptability of the home BP telemonitoring device among community-dwelling senior citizens with hypertension. Previous research has shown that 65% of the population aged ≥65 years would like to keep up-to-date with technological advancements, which includes health apps [26]; however, at present, there is a dearth of information on the unique needs and demands of senior citizens using medical devices at home [27]. In line with the results of a research conducted by Ehmen et al in which they concluded that mobile devices for measuring heart rate and electrocardiography were well accepted by participants aged ≥55 years [27], we hypothesized that participating senior citizens in this study would rate the home BP telemonitoring device as user friendly, acceptable, and useful for hypertension management. This hypothesis was supported by the study findings. However, a few issues were identified and potential solutions were explored in order to optimize device functionality and its successful future use.

One unique aspect of our study in comparison to prior studies is the advanced age of the participants (average age, 86 years). However, despite this difference, it is worth noting that our findings of high patient acceptability are consistent with the existing qualitative literature assessing patient views on BP telemonitoring systems [28-31]. Other studies have also included care providers in the qualitative assessment and have reported that while caregivers are generally supportive of the concept, they are concerned about the time required to view and act on telemonitored readings [30,31]. This underscores the need to develop systems and protocols that minimize provider time and replace (instead of adding to) existing workloads.

### User-Friendliness of the Device

In this study, all participants described the device as simple and straightforward to use. Specifically, the start button was large enough and easy to engage and the display screen was legible, even for those with visual impairments. This demonstrates that the design of this home BP telemonitoring is suitable for senior citizens of advanced age. Device acceptability and usability was enhanced by building a telemonitoring system that depended only on activating the home BP monitor. The remainder of the data transmission performed through communication between the monitor and the hub, and the hub and the cloud, was performed automatically. The fact that the monitor itself has been designed according to regulatory requirements and standards enhanced the probability that the users would find it usable and acceptable, as long as they were not required to perform additional steps besides activating a measurement.

Conversely, all participants identified one or more difficulties related to using the device. Specific issues were noted regarding the design and application of the BP cuff component. For example, it was described as being too large, having cumbersome extra material when pulled tight, and being difficult to place and secure with one hand. These concerns may be related to the features of normal, age-related decline in psychomotor skills, including dexterity and hand-eye coordination [32]; however, it is critical to optimize the design by addressing these concerns to overcome the barriers to implementation and successful adoption of home telemonitoring technology.

Apart from the BP cuff component, participants noted that error messages on the display screen were confusing, and one participant did not know how to manage the device when the batteries ran out. While these issues are seen as normal features, albeit limitations, of current technology, older adults are more affected by the distracting context, including unfamiliar or unexpected error messages, making it challenging to perform concurrent tasks, including changing batteries at the same time as taking a BP measurement [32]. These challenges are related to cognitive load, and previous research has suggested that an initial facilitated learning experience, supplemented with support materials, can be used to train senior citizens in proper use and functioning of the device and to resolve any potential errors or issues that may arise [27].

An issue that arose that was peripheral to the BP device itself was the lack of understanding of the wireless technology required to enable the telemonitoring aspect of this system. Six of 7 participants had little awareness of the relevant technology. Participants knew, in general, that the hub assisted with the operation of the telemonitoring system, but they were not clear on specific functions and did not touch or interfere with these components during their week of using the device. This finding is not surprising as consumers often lack understanding of the inner workings of commonly used technologies such as mobile phones and computers, but this lack of knowledge does not preclude the use of these devices. In fact, rather than having an intimate understanding of how the technology works, it is more important that users have access to assistance with troubleshooting if the device is not working properly. Thus, it is important that telemonitoring system vendors provide such assistance.

### Acceptability and Usefulness of the Device

As proxies for the acceptability and usefulness of the device, participants were asked if they would use the device in the future and if they would recommend this device to a friend. All 7 participants indicated that they would recommend this device to a friend in need of regular BP monitoring. The majority (n=6) indicated a willingness to use this device in the future if their health status required regular BP monitoring.

### Recommendations for Future Developments

It is worthwhile noting two specific factors that may influence future, appropriate use of this home BP telemonitoring device. First, 5 of 7 participants indicated some difficulty knowing when and how often to take measurements with the device. This could be addressed in both high- and low-tech approaches. For example, a high-tech solution could be a programmable device feature of either an audible signal or visual aid when it is time to take BP measurements. This is the advantage that mobile phone–connected strategies provide; however, these are not appropriate for populations with limited technology literacy. A low-tech solution could be to provide written, supporting information, like a daily calendar containing these details. For 5 out of 7 of participants, detailed instructions were provided as part of the “study pack”; this may indicate that the provided information was not formatted optimally to disseminate operational instruction. The high-tech improvement (ie, a signal from the device) was suggested by a participant during the interview and would likely be acceptable to users.

Second, a number of participants indicated uncertainty about whether they were using the device properly. Other than the potential for an error message on the display screen, the device did not have the capacity to provide patients with feedback as to whether the device was properly positioned and secured for optimal readings or if the readings were being properly transmitted through the wireless connection. This concern was noted by a participant when asked about suggestions for improvement. Additionally, there was no explanation of the error messages. When these warnings occurred, participants repeatedly attempted to take additional BP readings until the error message disappeared. It could be worthwhile to explore whether real-time feedback could be incorporated into the machine, which could include a written message on the display, an auditory message (voice, beep, etc) from the device, or a sensory message (squeeze, vibration, etc) in the cuff. This functionality may be especially important for successful, long-term use as the majority of participants lived alone and did not have any caregiver assistance to use the device.

### Conclusion

Overall, the home BP telemonitoring device had very good usability and acceptability among community-dwelling senior citizens with hypertension. This was illustrated by all participants indicating willingness to recommend this device to a friend and 6 of 7 participants stating they would use this device in the future. Additionally, all participants found the device to be simple and straightforward to use. To enhance its long-term use, a few improvements were noted that may mitigate some of the relatively minor challenges encountered by the target population.

## Appendix

Multimedia Appendix 1 Semistructured interview guide.

## Abbreviations

BP: blood pressure
RCT: randomized controlled trial
SIM: Subscriber Identity Module

## References

[1] (2018). World Health Organization.

[2] (2008). Statistics Canada.

[3] Franklin SS (2012). Elderly hypertensives: how are they different?. J Clin Hypertens (Greenwich).

[4] Aronow WS, Fleg JL, Pepine CJ, Artinian NT, Bakris G, Brown AS, Ferdinand KC, Forciea MA, Frishman WH, Jaigobin C, Kostis JB, Mancia G, Oparil S, Ortiz E, Reisin E, Rich MW, Schocken DD, Weber MA, Wesley DJ, Harrington RA, ACCF Task Force (2011). ACCF/AHA 2011 expert consensus document on hypertension in the elderly: a report of the American College of Cardiology Foundation Task Force on Clinical Expert Consensus Documents. Circulation.

[5] Staessen JA, Gasowski J, Wang JG, Thijs L, Den HE, Boissel JP, Coope J, Ekbom T, Gueyffier F, Liu L, Kerlikowske K, Pocock S, Fagard RH (2000). Risks of untreated and treated isolated systolic hypertension in the elderly: meta-analysis of outcome trials. Lancet.

[6] Lewington S, Clarke R, Qizilbash N, Peto R, Collins R, Prospective Studies Collaboration (2002). Age-specific relevance of usual blood pressure to vascular mortality: a meta-analysis of individual data for one million adults in 61 prospective studies. Lancet.

[7] Allen M, Kelly K, Fleming I (2013). Hypertension in elderly patients: recommended systolic targets are not evidence based. Can Fam Physician.

[8] Cushman WC, Evans GW, Byington RP, Goff DC, Grimm RH, Cutler JA, Simons-Morton DG, Basile JN, Corson MA, Probstfield JL, Katz L, Peterson KA, Friedewald WT, Buse JB, Bigger JT, Gerstein HC, Ismail-Beigi F, ACCORD Study Group (2010). Effects of intensive blood-pressure control in type 2 diabetes mellitus. N Engl J Med.

[9] Tinetti ME, Han L, Lee DSH, McAvay GJ, Peduzzi P, Gross CP, Zhou B, Lin H (2014). Antihypertensive medications and serious fall injuries in a nationally representative sample of older adults. JAMA Intern Med.

[10] Leung AA, Daskalopoulou SS, Dasgupta K, McBrien K, Butalia S, Zarnke KB, Nerenberg K, Harris KC, Nakhla M, Cloutier L, Gelfer M, Lamarre-Cliche M, Milot A, Bolli P, Tremblay G, McLean D, Tobe SW, Ruzicka M, Burns KD, Vallée M, Prasad GVR, Gryn SE, Feldman RD, Selby P, Pipe A, Schiffrin EL, McFarlane PA, Oh P, Hegele RA, Khara M, Wilson TW, Penner SB, Burgess E, Sivapalan P, Herman RJ, Bacon SL, Rabkin SW, Gilbert RE, Campbell TS, Grover S, Honos G, Lindsay P, Hill MD, Coutts SB, Gubitz G, Campbell NRC, Moe GW, Howlett JG, Boulanger J, Prebtani A, Kline G, Leiter LA, Jones C, Côté A, Woo V, Kaczorowski J, Trudeau L, Tsuyuki RT, Hiremath S, Drouin D, Lavoie KL, Hamet P, Grégoire JC, Lewanczuk R, Dresser GK, Sharma M, Reid D, Lear SA, Moullec G, Gupta M, Magee LA, Logan AG, Dionne J, Fournier A, Benoit G, Feber J, Poirier L, Padwal RS, Rabi DM, Hypertension Canada (2017). Hypertension Canada's 2017 Guidelines for Diagnosis, Risk Assessment, Prevention, and Treatment of Hypertension in Adults. Can J Cardiol.

[11] Omboni S, Ferrari R (2015). The role of telemedicine in hypertension management: focus on blood pressure telemonitoring. Curr Hypertens Rep.

[12] Wood PW, Boulanger P, Padwal RS (2017). Home Blood Pressure Telemonitoring: Rationale for Use, Required Elements, and Barriers to Implementation in Canada. Can J Cardiol.

[13] Omboni S, Gazzola T, Carabelli G, Parati G (2013). Clinical usefulness and cost effectiveness of home blood pressure telemonitoring: meta-analysis of randomized controlled studies. J Hypertens.

[14] Ninomiya T, Perkovic V, Turnbull F, Neal B, Barzi F, Cass A, Baigent C, Chalmers J, Li N, Woodward M, MacMahon S, Blood Pressure Lowering Treatment Trialists' Collaboration (2013). Blood pressure lowering and major cardiovascular events in people with and without chronic kidney disease: meta-analysis of randomised controlled trials. BMJ.

[15] Rabi DM, Padwal R, Tobe SW, Gilbert RE, Leiter LA, Quinn RR, Khan N, Canadian Hypertensive Education Program, Canadian Diabetes Association (2013). Risks and benefits of intensive blood pressure lowering in patients with type 2 diabetes. CMAJ.

[16] Padwal R, McAlister FA, Wood PW, Boulanger P, Fradette M, Klarenbach S, Edwards AL, Holroyd-Leduc JM, Alagiakrishnan K, Rabi D, Majumdar SR (2016). Telemonitoring and Protocolized Case Management for Hypertensive Community-Dwelling Seniors With Diabetes: Protocol of the TECHNOMED Randomized Controlled Trial. JMIR Res Protoc.

[17] Sandelowski M (2000). Whatever happened to qualitative description?. Res Nurs Health.

[18] Sandelowski M (2010). What's in a name? Qualitative description revisited. Res Nurs Health.

[19] Vizri RA (1992). Refining the test phase of usability evaluation: how many subjects is enough. Human Factors.

[20] Morse J, Field PA (1995). Qualitative research methods for health professionals (2nd edition).

[21] QSR International Pty Ltd (2015). NVivo qualitative data analysis Software. version.

[22] Vaismoradi M, Turunen H, Bondas T (2013). Content analysis and thematic analysis: Implications for conducting a qualitative descriptive study. Nurs Health Sci.

[23] Sparker AC, Holloway I (2005). Narrative analysis exploring the whats and hows of personal stories. Qualitative Research in Health Care (1st edition).

[24] Braun V, Clarke V (2006). Using Thematic Analysis in Psychology. Qualitative Research in Psychology.

[25] Fereday J, Muir-Cochrane EC (2006). Demonstrating rigor using thematic analysis: A hybrid approach of inductive and deductive coding and theme development. Int J Qual Methods.

[26] Narasimha S, Madathil KC, Agnisarman S, Rogers H, Welch B, Ashok A, Nair A, McElligott J (2017). Designing Telemedicine Systems for Geriatric Patients: A Review of the Usability Studies. Telemed J E Health.

[27] Ehmen H, Haesner M, Steinke I, Dorn M, Gövercin M, Steinhagen-Thiessen E (2012). Comparison of four different mobile devices for measuring heart rate and ECG with respect to aspects of usability and acceptance by older people. Appl Ergon.

[28] Ovaisi S, Ibison J, Leontowitsch M, Cloud G, Oakeshott P, Kerry S (2011). Stroke patients' perceptions of home blood pressure monitoring: a qualitative study. Br J Gen Pract.

[29] Koopman RJ, Wakefield BJ, Johanning JL, Keplinger LE, Kruse RL, Bomar M, Bernt B, Wakefield DS, Mehr DR (2014). Implementing home blood glucose and blood pressure telemonitoring in primary care practices for patients with diabetes: lessons learned. Telemed J E Health.

[30] Abdullah A, Liew SM, Hanafi NS, Ng CJ, Lai PS, Chia YC, Loo CK (2016). What influences patients' acceptance of a blood pressure telemonitoring service in primary care? A qualitative study. Patient Prefer Adherence.

[31] Hanley J, Ure J, Pagliari C, Sheikh A, McKinstry B (2013). Experiences of patients and professionals participating in the HITS home blood pressure telemonitoring trial: a qualitative study. BMJ Open.

[32] Kaufman DR, Patel VL, Hilliman C, Morin PC, Pevzner J, Weinstock RS, Goland R, Shea S, Starren J (2003). Usability in the real world: assessing medical information technologies in patients' homes. J Biomed Inform.

