# Supplementary material for: Usability and Acceptability of a Home Blood Pressure Telemonitoring Device Among Community-Dwelling Senior Citizens With Hypertension: Qualitative Study
Source: JMIR Aging. 2018 Jul 24;1(2):e10975. doi: 10.2196/10975 (PMC6716488; doi:10.2196/10975)
Supplement: Multimedia Appendix 1 [file aging_v1i2e10975_app1.pdf]

Multimedia Appendix 1.

Semistructured interview guide.

Good morning or afternoon. Thank you for taking the time to meet with me. I would like to ask you several questions about your experience using the blood pressure monitoring device. I am recording our conversation to ensure that we have an accurate summary of your opinions. All the information we collect will be kept confidential. You may refuse to answer any questions or leave the interview at any time. Do you have any questions before we begin? Please feel free to ask questions at any time during the interview. Let's get started;

1. Tell me about your experience using the blood pressure monitor over the past [length of time with device].

2. Was the blood pressure monitor simple to use?

3. Was there anything difficult about using it?
   Prompts: Tell me more. How was it difficult?

4. Did you ever encounter a problem when using the device?

5. Let's walk through how you used the device to measure your blood pressure. Can you please explain to me as we go what you did at each step?
   Prompts: What is the first thing you did? What did you do next? Then what?

6. Did you need any additional help to use the blood pressure monitor?
   Prompts: Tell me more. For example, did you read the written instructions or ask for someone for help? When did you need help?

7. How did you feel about measuring your blood pressure at home with this device?
   Prompts: Did you like it? Did you dislike it? Why? Why not?

8. Would you use the blood pressure monitor in the future?
   Prompt: Why or why not?

9. Would you recommend this blood pressure monitor to someone else?
   Prompt: Why or why not?

10. Is there anything else you would like to tell me about your experience with this blood pressure monitor?

Thank you for your thoughtful responses to my questions.
